# The Web-Based Uprise Program for Mental Health in Australian University Students: Protocol for a Pilot Randomized Controlled Trial

**DOI:** 10.2196/21307

**Published:** 2020-12-04

**Authors:** Karra D Harrington, Robert Eres, Michelle H Lim

**Affiliations:** 1 Iverson Health Innovation Research Institute Swinburne University of Technology Hawthorn Australia; 2 Center for Healthy Aging The Pennsylvania State University University Park, PA United States; 3 Centre for Mental Health Swinburne University of Technology Hawthorn Australia

**Keywords:** digital technology, e-mental health, university students, mental health, randomized controlled trial

## Abstract

**Background:**

University students are vulnerable to poor mental health, psychological distress, and loneliness relative to nonuniversity student peers. However, the rate of seeking mental health treatment among university students is low. Web-based psychological interventions may provide an opportunity for supporting vulnerable university students who are unlikely to otherwise seek support.

**Objective:**

The aim of this study is to examine the feasibility, acceptability, safety, and efficacy of an existing web-based transdiagnostic cognitive behavioral therapy (CBT) mental health program for use among Australian university students.

**Methods:**

This is a pilot randomized controlled trial comparing a self-directed web-based CBT mental health program with a waitlist control. The self-directed modules will be augmented with optional webchat or telephone coaching with a therapist. The recruitment target is 70 university students who do not present with a clinical mental health disorder. Allocation will be made in a 1:1 ratio and will occur after the initial baseline assessment. Assessments will be completed at baseline, upon completion of a 4-week waitlist (waitlist group only), upon completion of the program, and at 3 months after completion of the program.

**Results:**

The trial was funded in June 2018, and the protocol was approved by the Swinburne University Human Research Ethics Committee in September 2018. Recruitment commenced in October 2018, with the first participant allocated in November 2018. A total of 70 participants were recruited to the trial. The trial recruitment ceased in June 2019, and data collection was finalized in December 2019. We expect the final data analysis to be completed by November 2020 and results to be published early in 2021. The primary outcomes are feasibility, acceptability, safety, and symptoms of depression, anxiety, and stress. The secondary outcomes are psychological wellbeing, quality of life, loneliness, self-reported physical health status, emotion regulation, and cognitive and mindfulness processes.

**Conclusions:**

The acceptability, feasibility, safety, and efficacy of a web-based mental health program in university students will be evaluated. Web-based mental health programs offer the opportunity to engage university students who may be reluctant to seek support through traditional face-to-face mental health services, and the transdiagnostic approach of the program has the potential to address the breadth of mental health concerns of university students.

**Trial Registration:**

Australian New Zealand Clinical Trial Registry ACTRN12618001604291; https://www.anzctr.org.au/Trial/Registration/TrialReview.aspx?ACTRN=12618001604291

**International Registered Report Identifier (IRRID):**

DERR1-10.2196/21307

## Introduction

Young adults aged 16 to 24 years report the highest prevalence of mental health disorders of any age group in the Australian population [1]. University students within this age group are particularly vulnerable to experience poor mental health and psychological distress relative to nonuniversity student peers [2,3]. The most commonly identified mental health issues for university students are depression, anxiety, and stress [4,5]. University students further report unhelpful psychological processes, including negative/critical thinking, self-blame, worry, pessimism, confusion, thoughts of death, loss of confidence, and poor self-esteem [5]. Changes in social relationships specifically may exacerbate the development of mental health symptoms, especially for those who are socially isolated (eg, those living alone or off-campus and those who have migrated for study [3,6,7]). This is consistent with research findings that earlier loneliness predicts more severe depression, social anxiety, and paranoia at a later time [8], and that living away from home for young people may increase their risk of loneliness and poor mental health [9,10].

Despite the high rates of mental health disorders (including anxiety and depression [4,11]) and high psychological distress among university students, the rate of seeking mental health treatment is low [12]. Only about one-third of students who report psychological distress also report accessing on-campus counselling services [13]. According to qualitative reports from university students, stigma related to mental ill health remains a major factor in preventing them from seeking support or treatment for mental health symptoms [5]. Given the reluctance of university students to seek mental health treatment despite high levels of psychological distress, alternate intervention methods to enhance mental health in this population are required. One approach that shows promise to address mental health symptoms and psychological distress for university students is the use of e-mental health or online psychological interventions [14-18]. University students report a willingness to use online mental health resources [19] and perceive multiple benefits of doing so, such as avoidance of stigma, immediacy and ease of access, anonymity and privacy, and greater sense of control over the help-seeking journey [20,21]. Furthermore, students who report being least likely to seek support through an on-campus counselling service or mental health service are most likely to indicate that they would access a web-based student wellbeing program [22]. Thus, online mental health programs and interventions provide a useful approach for supporting vulnerable university students who are unlikely to otherwise seek support.

Uprise (Uprise Services [23]) is an existing web-based mental health program that applies a transdiagnostic cognitive behavioral therapy (CBT) approach for reducing mental health symptoms and improving psychological wellbeing. CBT is considered to be the current gold standard for psychological intervention and has strong evidence for efficacy in reducing symptoms across a range of mental health disorders [24,25]. There is some evidence that CBT-based interventions also influence psychological processes underlying the manifestation of symptoms in many mental health disorders, including correcting habitual thinking errors, reducing biases in attention or memory, and improving emotion regulation [26,27]. The Uprise program comprises a series of self-directed modules that address mindset, values, mindfulness, and stress management. The self-directed modules are augmented with optional telephone or webchat coaching with a trained mental health professional. The option for the self-directed modules to be augmented with coaching support is consistent with results from previous surveys of user preferences indicating that format flexibility is important for the delivery of effective e-mental health interventions [16,28]. Previous trials of similar web-based programs have demonstrated this self-directed CBT-based approach to be effective in reducing symptoms of generalized anxiety, panic disorder, social phobia, obsessive compulsive disorder, and posttraumatic stress disorder in clinically diagnosed adult and student populations [18,29-31]. However, the potential for these interventions to change psychological processes was not examined in these prior studies, and many studies did not present acceptability outcomes.

The primary aim of this trial is to evaluate the acceptability, feasibility, safety, and efficacy of the Uprise program in reducing mental health symptoms among Australian university students. The secondary aims include evaluating the holistic impact of the Uprise program and investigating whether the Uprise program influences psychological processes, such as emotion regulation, cognitive appraisals, and mindfulness. This trial extends on the outcomes of previous trials of similar web-based CBT interventions by examining the broader holistic impact of the intervention rather than focusing only on symptom reduction.

## Methods

### Trial Design

**Figure 1:**
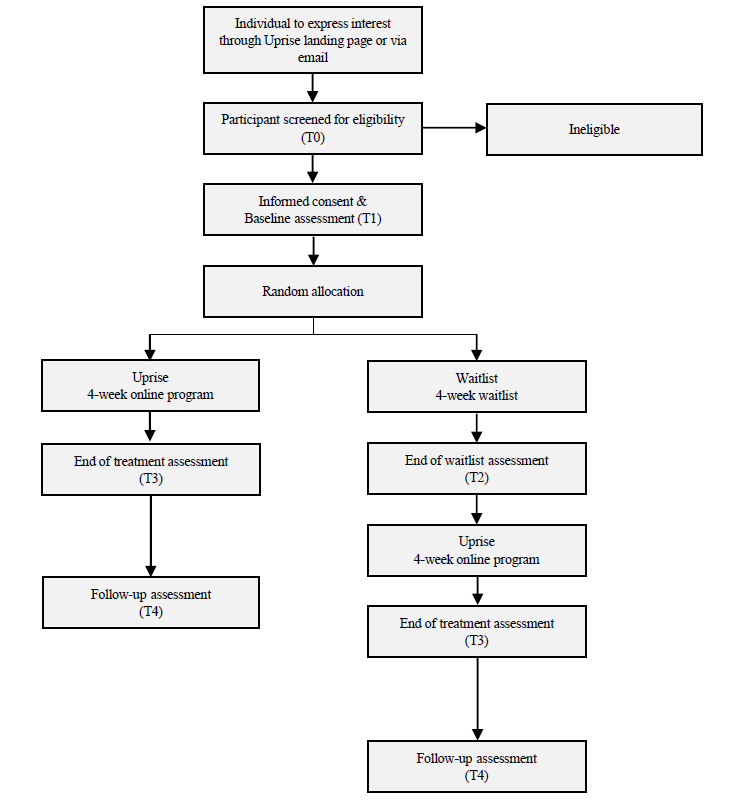
Consolidated Standards of Reporting Trials (CONSORT) diagram showing the trial design.

The study will be a pilot randomized waitlist-controlled trial, with two parallel groups, using a 1:1 allocation ratio. Waitlist was selected as the control condition to enable comparison of the Uprise program to students’ usual daily life, while ensuring that all participants had the opportunity to access the Uprise program. Figure 1 presents the study design.

Participants will initially undergo screening for eligibility via telephone interview (T0). Eligible participants will be invited to complete the baseline assessment (T1). Informed consent will be obtained at the baseline assessment. Following the baseline assessment, each consenting participant will be randomized to either the Uprise program or waitlist for the following 4 weeks. Those allocated to the Uprise program will be given access to the program and asked to complete one module per week across the following 4-week period. At the end of the program, these participants will be invited to complete an end-of-treatment assessment (T3). Participants allocated to waitlist will be asked to continue with their usual activities across the following 4-week period, at the end of which they will be invited to complete an end-of-waitlist assessment (T2). Waitlist participants will then be given access to the Uprise program and asked to complete one module per week for the following 4 weeks, at the end of which they will be invited to complete an end-of-treatment assessment (T3). Participants will also be invited to complete a semistructured interview at the end-of-treatment (T3) assessment regarding their experience with the intervention. The interview will be recorded and later transcribed for qualitative analysis. All participants will be invited to complete a follow-up assessment (T4) 3 months after their end-of-treatment (T3) assessment.

The Consolidated Standards of Reporting Trials (CONSORT) flow diagram of the study procedure is shown in Figure 1. The protocol was designed in accordance with the Standard Protocol Items: Recommendations for Interventional Trials (SPIRIT) and good clinical practice (GCP) guidelines (see Multimedia Appendix 1 and Multimedia Appendix 2 for relevant checklists).

### Study Setting

This trial will be coordinated from Swinburne University of Technology in Melbourne, Australia, and will aim to include students from multiple universities across Australia. Trial assessments will be conducted remotely via telephone and online questionnaires. The trial intervention will also be administered remotely via the Uprise web platform and phone app, with optional coaching calls conducted via either telephone or webchat according to participant preference.

### Sample Size

Seventy participants will be recruited to the trial. Power analysis was conducted using G Power 2.0 for the mental health primary outcome variables (social anxiety and depression), with estimates based on published means and standard deviations and reported effects of similar interventions [32]. The analysis indicated that a minimum of 27 to 45 participants per group would be required to detect moderate changes with 80% power in social anxiety and depression (standardized Cohen *d* of 0.43 to 0.57). Owing to funding constraints, only 35 participants will be recruited to each arm.

### Recruitment and Enrolment

Participants will be recruited from Australian universities via social media advertisements, flyers, and student newsletter articles. At Swinburne University, students who are identified as being “at risk” of dropping out of the university owing to mental health concerns will also be emailed and invited to participate in the study. Identification of “at risk” students will be completed by Swinburne University student wellbeing officers according to whether a student did not receive a passing grade for one or more subjects in the previous semester. Student names and email addresses will be shared with the researchers for the purpose of targeted trial recruitment. No other details will be shared with the research team. This targeted recruitment approach has been included to reach vulnerable students who may not have otherwise been motivated to engage in a research study, as well as to emulate potential pathways to accessing the Uprise program if it were to be rolled out as a wellbeing program at universities.

Participants will be invited to register their interest in the study via direct email to the research team or by registering their interest on the study landing page. Some participants may directly comment on social media posts, in which case we will direct them to email the research team or visit the trial landing page. The research team will then email potential participants an information sheet and informed consent form. Once participants have provided their contact details and a preferred contact time, researchers will telephone participants to conduct eligibility screening. The study sponsors will not have access to any identifiable recruitment information.

### Eligibility Criteria

Participants meeting the following criteria will be eligible for the study: (1) aged 17 to 26 years; (2) currently enrolled as a student at an Australian university; (3) competent in English reading and comprehension; and (4) able to receive phone or video conference calls or visit the research center for assessments. The exclusion criteria are as follows: (1) self-reported acute or distressing clinical psychiatric symptoms in the past month (ie, symptoms that the individual reported experiencing as distressing or interfering with their usual daily activities); (2) psychiatric hospitalization in the past month; (3) self-report of any level of suicidality risk; (4) self-report of moderate or severe levels of distress (score equal to or more than 25 on the Kessler Psychological Distress Scale) during screening assessment; (5) self-report of any level of risk of harm to others; and (6) self-report of any level of risk of damage to objects or property.

Participants who report mental health symptoms of a severity that deems them ineligible for the trial will be referred to appropriate mental health services. This is to ensure that these participants receive the appropriate level of care to address the severity of mental health symptoms, as the Uprise program is not designed to address severe symptoms or suicidality. To ensure equitable access to the trial and to minimize sampling bias, these participants will be given the option to undergo eligibility screening again at a later time and to participate in the trial should they meet criteria at this second screening.

### Allocation and Blinding

Participants confirmed to be eligible following the screening assessment (T0) will be invited to complete the baseline assessment (T1). Following the baseline assessment, participants will be subject to random allocation to either waitlist or treatment with the Uprise program. Block randomization will be applied in this study with a 1:1 allocation ratio using random block sizes of 4 and 6. The randomization sequence will be generated using the Sealed Envelope online randomization sequence generator [33]. Randomization and allocation of participants will be performed by a member of the trial research team (KDH). Allocation will occur within 7 days of the baseline assessment. Participants will be contacted to notify them of their allocation, with those allocated to the treatment group given instructions to access the Uprise program and those allocated to the waitlist group notified of the date for their next assessment (T2).

All outcome assessments will be completed online by participants. It is not possible to blind participants to their allocation owing to the nature of the trial design. The psychologists who provide “coaching” as part of the Uprise program will be blind to participant allocation.

### Materials

Table 1 presents the SPIRIT schedule of measures in this study. The primary outcomes will focus on the acceptability, feasibility, and safety of the Uprise program, as well as efficacy as indicated by the change in mental health symptoms (depression, anxiety, and stress). The secondary outcomes will include psychological wellbeing, quality of life, loneliness, physical health, emotion regulation, and cognitive and mindfulness processes.

Demographic variables will include age, gender, sexuality, ethnicity, work status, level of education, previously attempted university degrees, current university course load, postcode, and religion. The Lubben Social Network Scale-12 items (LSNS-12 [34]) will be used as a demographic assessment of the participants’ risk of social isolation. The Positive and Negative Affect Schedule-Trait (PANAS [35]) will be used as a measure of trait-level positive and negative affect.

**Table 1:** SPIRIT schedule of enrolment, interventions, and assessments.

Variable		Study period							
		Enrolment	Baseline	Postallocation				Close out	
				Waitlist	End of waitlist	Uprise	End of treatment	3-month follow-up	
Timepoint		T0	T1	N/A^a^	T2	N/A	T3	T4	
**Enrolment**									
	Eligibility screen	Yes	No	No	No	No	No	No	
	Informed consent	No	Yes	No	No	No	No	No	
	Allocation	No	Yes	No	No	No	No	No	
**Interventions**									
	Uprise program	No	No	No	No	Yes	No	No	
	Waitlist	No	No	Yes	No	No	No	No	
**Assessments**									
	Demographic form	No	Yes	No	No	Yes	No	No	
	UCLA-LS3^b^	No	Yes	No	Yes	No	Yes	Yes	
	CES-D^c^	No	Yes	No	Yes	No	Yes	Yes	
	SIAS^d^	No	Yes	No	Yes	No	Yes	Yes	
	DASS-21^e^	No	Yes	No	Yes	No	Yes	Yes	
	PANAS^f^	No	Yes	No	Yes	No	Yes	Yes	
	K10^g^	Yes	No	No	Yes	No	Yes	Yes	
	LSNS-12^h^	No	Yes	No	Yes	No	Yes	Yes	
	ERQ^i^	No	Yes	No	Yes	No	Yes	Yes	
	FFMQ^j^	No	Yes	No	Yes	No	Yes	Yes	
	CBPQ^k^	No	Yes	No	Yes	No	Yes	Yes	
	BCIS^l^	No	Yes	No	Yes	No	Yes	Yes	
	PWB-42^m^	No	Yes	No	Yes	No	Yes	Yes	
	AQoL-8D^n^	No	Yes	No	Yes	No	Yes	Yes	
	PHQ^o^	No	Yes	No	Yes	No	Yes	Yes	
	SF-12^p^	No	Yes	No	Yes	No	Yes	Yes	
	WHO-5^q^	No	No	No	No	Yes	No	No	
	PSS-4^r^	No	No	No	No	Yes	No	No	
	Modified SDS^s^	No	No	No	No	Yes	No	No	
	Acceptability measure	No	No	No	No	Yes	No	No	
	Qualitative interview	No	No	No	No	No	Yes	No	

^a^N/A: not applicable.

^b^UCLA-LS3: UCLA Loneliness Scale Version 3.

^c^CES-D: Centre for Epidemiological Studies-Depression.

^d^SIAS: Social Interaction Anxiety Scale.

^e^DASS-21: Depression, Anxiety, and Stress Scale-21 items.

^f^PANAS: Positive and Negative Affect Schedule-Trait and State versions.

^g^K10: Kessler Scale of Distress-10 items.

^h^LSNS-12: Lubben Social Network Scale-12 items.

^i^ERQ: Emotion Regulation Questionnaire.

^j^FFMQ: Five Facet Mindfulness Questionnaire.

^k^CBPQ: Cognitive Behavioral Processes Questionnaire.

^l^BCIS: Beck Cognitive Insight Scale.

^m^PWB-42: Psychological Well-Being Scale-42 items.

^n^AQoL-8D: Assessment of Quality of Life-8 Dimensions.

^o^PHQ: Physical Health Questionnaire.

^p^SF-12: Short-Form Health Survey-12 items.

^q^WHO-5: World Health Organization-Five Well-Being Index.

^r^PSS-4: Perceived Stress Scale-4 items.

^s^SDS: Sheehan Disability Scale.

### Primary Outcomes

#### Acceptability, Feasibility, and Safety of the Uprise Program

A self-report scale created by the developers of the Uprise program and designed to measure participant satisfaction with the program will be used to determine the acceptability of the program to participants. Participants will be asked to rate out of 10 how satisfied they were with each of the program modules and coaching calls, as well as how likely they are to recommend Uprise to friends or family. Attrition rates from the trial will also be used as indicators of acceptability. Themes identified from the qualitative interviews conducted at the end-of-treatment (T3) assessment will also be used to determine acceptability of the intervention. The interview will include questions regarding the participants’ experiences and preferences regarding the different modules of the Uprise program. Feasibility will be assessed by the proportion of interested people who complete the baseline assessment, the attrition rate across both groups, and the proportion of participants who complete the four core Uprise program modules within the 6-week intervention period. Safety will be determined according to the number of adverse events occurring across the trial period.

#### Mental Health

Psychological distress will be measured with the Kessler Scale of Distress-10 items (K10 [36]). Depression will be measured with the Centre for Epidemiological Studies-Depression (CES-D [37]) and depression subscale of the Depression Anxiety Stress Scale-21 items (DASS-21 [38]). Anxiety will be measured with the anxiety subscale of the DASS-21, and social anxiety will be measured with the 20-item Social Interaction Anxiety Scale (SIAS [39,40]).

#### Stress

The Perceived Stress Scale (PSS-4 [41]) and stress subscale of the DASS-21 will be used to measure stress. The Modified Sheehan Disability Scale (modified SDS [42]) will be used to measure the effects of mental illness, physical illness, and stress on work and study.

### Secondary Outcomes

#### Wellbeing and Quality of Life

The Psychological Well-Being Scale-42 items (PWB-42 [43,44]), the World Health Organization-Five Well-Being Index (WHO-5 [45]), and the Assessment of Quality of Life-8 Dimensions (AQoL-8D [46]) will be used to measure global wellbeing and life satisfaction. The PWB measures psychological wellbeing across the following dimensions: autonomy, positive relations with others, environmental mastery, personal growth, purpose in life, and self-acceptance. The AQoL-8D measures wellbeing across the following dimensions: independent living, happiness, mental health, coping, relationships, self-worth, pain, and senses.

#### Loneliness

The UCLA Loneliness Scale-Version 3 (UCLA-LS [47]) is a 20-item self-report measure and consists of both positively and negatively worded items that assess loneliness (eg, How often do you feel that you are no longer close to anyone?).

#### Physical Health

Question 1 from the Short-Form Health Survey-12 items (SF-12 [48,49]) will be used as a single measure of health, and it asks participants to rate their perception of their overall health quality. The Physical Health Questionnaire (PHQ [50]) is a 14-item brief self-report scale of physical health symptoms.

#### Emotion Regulation, Cognitive, and Mindfulness Processes

The Emotion Regulation Questionnaire (ERQ [51]) will be used to measure participants’ tendency to use cognitive reappraisal and expressive suppression strategies to regulate their emotions. Activated affect in the past week, measured with the Positive and Negative Affect Schedule-State (PANAS-State [35]), will be used as a secondary indicator of emotion regulation. The Cognitive Behavioral Processes Questionnaire (CBPQ [52]) will be used to measure internal and external cognitive and behavioral maintenance processes. The Beck Cognitive Insight Scale (BCIS [53]) will be used to measure participants’ self-reflectiveness and self-certainty in their interpretations of their experiences. The Five Facet Mindfulness Questionnaire (FFMQ [54]) is a measure of mindfulness and comprises the following five subscales: observing, describing, acting with awareness, nonjudging of experience, and nonreactivity to experience.

### Planned Intervention

The Uprise program will be made accessible to participants as both an app and a website. Participants will complete all intervention activities online at a time and location that is convenient for them. Activities will include a series of self-directed modules and optional coaching sessions with a psychologist or counsellor.

Participants will complete four core modules over 4 to 6 weeks, with an additional six optional modules. Each module consists of a short introductory video and a series of 1 to 6 additional videos or exercises. The module length ranges from 6 to 28 minutes. The modules are mindset, personal values, mindfulness, and stress management (outlined in Table 2). To support adherence to the intervention protocol, a member of the research team will contact participants weekly to check on their progress and address any technical issues.

**Table 2:** Outline of the Uprise module content.

Module		Content
**Core modules**		
	Mindset	Identifying unhelpful thinking styles and changing unhelpful thoughts.
	Personal values	Identifying values system, making behavioral choices based on values system, and scheduling activities.
	Mindfulness	Developing mindfulness skills to pay attention to and observe thoughts instead of trying to control and change them.
	Stress management	Developing stress reduction and relaxation breathing skills.
**Optional modules**		
	Helping others	Skills for managing relationships with mental health in mind.
	Perspective taking	Learning to understand the perspectives of others.
	Advanced mindset	Advanced skills in retraining thinking related to stress, guilt, metacognition, and beliefs.
	Improving sleep	Strategies to improve sleep habits.
	Advanced mindfulness	Advanced skills in awareness and mindfulness.

#### Optional Coaching Sessions

The Uprise program also provides participants with in-website links to telephone or online coaching sessions with a trained mental health professional at any point during the program. Participants will be able to choose from a list of psychologists and counsellors to select their “coach” and schedule weekly 30-minute telephone or webchat sessions with their coach. It will be up to the participant to decide whether to engage with this aspect of the program.

### Waitlist Condition

It will be clearly outlined to participants that the trial involves a waitlist control prior to enrolment, and informed consent will include agreement to undergo a 4-week waitlist period and an additional assessment prior to commencing the Uprise program. Waitlist participants will be instructed to continue with their usual activities throughout the waitlist period.

### Planned Data Analysis

Summary data will be provided to describe the number of participants who registered interest in the trial, the number deemed ineligible for the trial and why, and the number who completed each stage of the trial.

Data will be analyzed on an intention-to-treat basis (ie, including all enrolled participants regardless of whether they completed the trial). Acceptability, feasibility, and safety will be evaluated using descriptive statistics. Qualitative interviews will be analyzed using the six-step approach by Braun and Clarke [55] for qualitative data analysis to identify and develop themes from the data to further inform about the acceptability of the intervention to participants.

Results will be summarized as means and standard deviations (or median and range/percentage and number as appropriate) for both groups at baseline (T0) and for the relevant group at the end of waitlist (T2)/end of treatment (T3) for all outcome measures. For both groups, this will demonstrate their results at baseline and again 4 weeks after baseline.

Additionally, the *t* test and chi-square test will be used to evaluate baseline group differences in all demographic and outcome measures. This will be performed to compare the waitlist and intervention groups, and to compare those who drop out from the trial to those who complete the trial. This will enable identification of potential baseline moderator variables or factors that contribute to attrition that can be included as covariates in subsequent analyses.

Multilevel linear mixed effects modeling (MLM) will be used to assess acute and long-term treatment effects in each of the outcome measures. MLM is a regression-based approach that is robust to missing data and provides a more powerful means of analyzing longitudinal data than the ANOVA family of analyses [56]. MLM is robust to missing data because it does not delete cases list-wise due to missing data, but instead includes all available data points for each participant. Missing data analysis will be conducted to determine whether data are missing completely at random. If it is identified that data are missing not at random, covariates will be added to all models to account for systematic variation related to missingness.

To assess short-term treatment effects, the trajectory of change in each of the outcomes will be examined from baseline (T1) to the end of waitlist (T2) for the waitlist group and from baseline (T1) to the end of treatment (T3) for the intervention group. The trajectory of change in each of the outcomes will be compared between groups. Each analysis will include time (fixed), group (fixed), and the fixed interaction between time and group (time × group) as predictors. A significant time × group interaction effect will indicate a differential rate of change in outcomes between the groups from baseline (T1) to the end of waitlist (T2)/end of treatment (T3).

To assess long-term treatment effects, the trajectory of change in outcomes from baseline (T1) to 3-month follow-up (T4) will be examined in the intervention group only. Sensitivity analysis will also be conducted to determine the trajectory of change from baseline (T1) to 3-month follow-up (T4) in the waitlist group only.

Secondary analyses will include per protocol analysis (only those who complete the four core modules within 6 weeks) and analyses controlling for moderator variables (eg, age, sex, baseline symptom severity, number of Uprise program modules completed, number of coaching calls completed, and number of days accessing the platform). We will report 95% confidence intervals for each test statistic.

### Data Monitoring and Management

#### Assessment Data

Data collected in this pilot randomized control trial will be managed in accordance with GCP guidelines. Restrictions to the data will be made such that only those listed as authors in this protocol, as well as the host institutes, will have access to the data. The host institutes’ access will be specific to audit and regulatory processes and will not be used for any purpose outside the scope of the trial registry.

All researchers will be provided training on assessment administration and scoring, as well as relevant ethics procedures and protocols for managing and storing data. Assessment data will be collected online via the Qualtrics survey platform [57] using a digital case report form. Data will be extracted to a secure data file and stored on a secure network. For each stage of data cleaning and analysis that is performed, a separate time-stamped computer file will be created and saved within an organized file system. To aid data quality, checks will include examination of recorded data for out-of-range values and data entry errors. Local ethics guidelines do not require a data monitoring committee for this type of trial. No interim analysis is anticipated.

#### Uprise Program Data

The Uprise program and online platform used in this study are existing clinical tools used in workplaces to support worker mental health. Uprise Services data security policies are fully compliant with Australian data protection and health record legislation [23]. Data collected via the Uprise online platform will be securely transferred to the research team via a 128-bit AES-encrypted data file. The password for the secure data file will be provided to researchers via telephone calls from a member of the Uprise Services team.

### Research Governance and Ethics

The trial will be administered by Swinburne University of Technology. The study has been approved by Swinburne University Human Research Ethics Committee (SUHREC; project 2018/205). The trial will be conducted in accordance with the Declaration of Helsinki, GCP guidelines, and the Australian National Statement on Ethical Conduct in Human Research [58]. Major protocol amendments will be submitted to SUHREC for review and approval and detailed in the trial registry. Full written informed consent will be obtained from participants at the time of the baseline assessment by a member of the research team. A copy of the consent form can be obtained by request from the corresponding author. There are no restrictions of the reporting findings of this trial. Trial results will be published in full in peer-reviewed literature.

### Serious Adverse Events

Any serious adverse events discovered by a research team member on the trial will be reported to the chief investigator Dr Michelle Lim and SUHREC. In the context of the trial, serious adverse events are defined as events that lead to participant death, are life-threatening, require inpatient hospitalization, or result in persistence of high disability/incapacity in accordance with the National Statement on Ethical Conduct in Human Research [58]. Any such events will be recorded and reviewed by the Chief Investigator to determine the likelihood of any relationship to the intervention, with action taken as appropriate. This may include referral to the Swinburne University Psychology Clinic or other health service for clinical treatment as required. All participants are provided with details for support services on the Participant Information Statement, including contact information for the Swinburne University Psychology Clinic and two crisis telephone lines.

### Trial Management Committee

A trial management committee will involve the named authors on this protocol as well as an independent academic not associated with the project. The project will be coordinated by the chief investigator (MHL) who will also collaborate with senior coinvestigators (KDH and RE) with regard to overseeing the trial. The chief investigator will ensure that each member of the trial management committee holds current GCP training and will be responsible for ensuring the safety of the participants and that the quality of the trial is not compromised.

## Results

The trial was funded in June 2018, and this protocol was approved by the Swinburne University Human Research Ethics Committee in September 2018. Recruitment commenced in October 2018, with the first participant allocated in November 2018. A total of 70 participants were recruited to the trial. The trial recruitment ceased in June 2019, and data collection was finalized in December 2019. We expect the final data analysis to be completed by November 2020 and results to be published early in 2021.

## Discussion

The Uprise program is derived from evidence-based psychological interventions; however, the efficacy of the program has not previously been formally evaluated. Therefore, the aim of the study is to determine the acceptability, feasibility, and safety of Uprise, and evaluate its efficacy in addressing mental health symptoms in university students. The trial extends on previous similar trials by also considering the holistic impact of the Uprise program and specifically evaluating changes in psychological processes that are relevant to the intervention. Digital platforms, such as the Uprise program, have the potential to engage university students who may be reluctant to engage traditional face-to-face services.

Digital or web-based psychological interventions offer an opportunity to support vulnerable university students who are unlikely to otherwise seek support. Young people often use digital tools to interact with peers [59] and, as such, are likely to find online mental health solutions acceptable [32]. In fact, those students who are least likely to access traditional mental health services report the greatest interest in accessing online student wellbeing programs [22]. Uprise is a transdiagnostic program that can be delivered via an app or a website and is based on the principles of CBT. The Uprise program is specifically designed to address mindset, personal values, mindfulness, and stress management skills. Given the breadth of mental health concerns and factors that contribute to psychological distress among university students, such a transdiagnostic approach to improving mental health has the potential to be beneficial for this population.

## Appendix

Multimedia Appendix 1 CONSORT eHEALTH Checklist V 1.6.1.

Multimedia Appendix 2 SPIRIT Checklist.

## Abbreviations

AQoL-8D: Assessment of Quality of Life-8 Dimensions
BCIS: Beck Cognitive Insight Scale
CBPQ: Cognitive Behavioral Processes Questionnaire
CBT: cognitive behavioral therapy
CES-D: Centre for Epidemiological Studies-Depression
DASS-21: Depression, Anxiety, and Stress Scale-21 items
ERQ: Emotion Regulation Questionnaire
FFMQ: Five Facet Mindfulness Questionnaire
GCP: good clinical practice
K10: Kessler Scale of Distress-10 items
LSNS-12: Lubben Social Network Scale-12 items
MLM: multilevel linear mixed effects modeling
PANAS: Positive and Negative Affect Schedule
PHQ: Physical Health Questionnaire
PSS-4: Perceived Stress Scale-4 items
PWB-42: Psychological Well-Being Scale-42 items
SDS: Sheehan Disability Scale
SF-12: Short-Form Health Survey-12 items
SIAS: Social Interaction Anxiety Scale
SUHREC: Swinburne University Human Research Ethics Committee
UCLA-LS: UCLA Loneliness Scale Version 3
WHO-5: World Health Organization-Five Well-Being Index
